# High-Performance CVD Bilayer MoS_2_ Radio Frequency Transistors and Gigahertz Mixers for Flexible Nanoelectronics

**DOI:** 10.3390/mi12040451

**Published:** 2021-04-16

**Authors:** Qingguo Gao, Chongfu Zhang, Kaiqiang Yang, Xinjian Pan, Zhi Zhang, Jianjun Yang, Zichuan Yi, Feng Chi, Liming Liu

**Affiliations:** 1 School of Electronic Information, University of Electronic Science and Technology of China, Zhongshan Institute, Zhongshan 528402, China; gqgemw@163.com (Q.G.); 201811022515@std.uestc.edu.cn (K.Y.); xinjpan@163.com (X.P.); zz001@zsc.edu.cn (Z.Z.); sdyman@uestc.edu.cn (J.Y.); yizichuan@zsc.edu.cn (Z.Y.); chifeng@semi.ac.cn (F.C.); limingliu@uestc.edu.cn (L.L.); 2 School of Information and Communication Engineering, University of Electronic Science and Technology of China, Chengdu 611731, China

**Keywords:** bilayer MoS_2_, CVD, high-frequency transistors, flexible electronics

## Abstract

Two-dimensional (2D) MoS_2_ have attracted tremendous attention due to their potential applications in future flexible high-frequency electronics. Bilayer MoS_2_ exhibits the advantages of carrier mobility when compared with monolayer mobility, thus making the former more suitable for use in future flexible high-frequency electronics. However, there are fewer systematical studies of chemical vapor deposition (CVD) bilayer MoS_2_ radiofrequency (RF) transistors on flexible polyimide substrates. In this work, CVD bilayer MoS_2_ RF transistors on flexible substrates with different gate lengths and gigahertz flexible frequency mixers were constructed and systematically studied. The extrinsic cutoff frequency (*f*_T_) and maximum oscillation frequency (*f*_max_) increased with reducing gate lengths. From transistors with a gate length of 0.3 μm, we demonstrated an extrinsic *f*_T_ of 4 GHz and *f*_max_ of 10 GHz. Furthermore, statistical analysis of 14 flexible MoS_2_ RF transistors is presented in this work. The study of a flexible mixer demonstrates the dependence of conversion gain versus gate voltage, LO power and input signal frequency. These results present the potential of CVD bilayer MoS_2_ for future flexible high-frequency electronics.

## 1. Introduction

Owing to their ultimate thin thickness, superior mechanical flexibility and highly tunable electronic performance, two-dimensional materials for flexible applications have attracted tremendous attention [1,2,3,4,5]. Flexible transistors and devices based on 2D materials with high-speed are easy integrated with mature silicon CMOS manufacturing systems and can extend future flexible electronic systems even further with remote wireless capabilities. Recently, numerous devices and circuits using graphene, black phosphorous (BP) and molybdenum disulfide (MoS_2_) were demonstrated for radio-frequency flexible electronics [6,7,8,9,10,11,12,13]. Graphene, which has an extraordinary carrier mobility of over 10,000 cm^2^/Vs and a high saturation velocity, has caused many researchers to realize high-performance flexible RF electronics with this extraordinary material. However, the current saturation of graphene transistors is poor due to the lack of bandgap of graphene [1,14]. Therefore, as a result, most graphene RF transistors may have a relatively lower *f*_max_, which limits power gain at high frequency. On the other hand, BP has a sizeable bandgap and moderate carrier mobility (1000 cm^2^/Vs), which makes it suitable for both low-power and high-speed flexible devices [13,15,16]. However, its poor reliability and lack of large-area high-quality growth capabilities are still the key technological barriers in BP high-frequency electronics [17].

Unlike graphene and BP, two-dimensional MoS_2_ with long-term air stability has a sizable bandgap, relative moderate carrier mobility and saturation velocity, and large-area growth capability. As an atomically thin semiconductor, 2D MoS_2_ have demonstrated great potential for use in high-frequency electronics, next-generation logic circuits and flexible electronics [9,18,19,20,21,22]. In 2014, radio-frequency transistors fabricated using exfoliated MoS_2_ demonstrated an extrinsic *f*_T_ of 2.1 GHz and *f*_max_ of 8 GHz on rigid Si/SiO_2_ substrates [22]. In the same year, exfoliated few-layer MoS_2_ RF transistors with self-aligned gates on rigid quartz and flexible polyimide substrates were reported [9]. The frequency responses of the self-aligned transistors on rigid quartz substrate showed an extrinsic *f*_T_ of 10.2 GHz and *f*_max_ of 14.5 GHz; a gigahertz inverter and amplifier were also demonstrated. Additionally, the frequency response of the self-aligned transistors on flexible PI substrate shown an extrinsic *f*_T_ and *f*_max_ of 4.7 and 5.4 GHz, respectively. Recently, Zhang et al. presented an flexible rectenna based on MoS_2_ high-frequency diodes with a cutoff frequency of 10 GHz [18]. Although these works have demonstrated the potential of high-frequency MoS_2_ devices, exfoliated MoS_2_ lack precise control of flake size and thickness, which hamper large-scale commercial manufacturing and applications [23,24].

Chemical vapor deposition (CVD) is a low-cost and important method to produce large-area, high-quality MoS_2_ film. High-quality uniform CVD monolayer MoS_2_ film larger than two inches has been growth by different groups [4,23,24,25]. In 2015, RF transistors based on CVD monolayer MoS_2_ with an extrinsic *f*_T_ of 2.8 GHz and *f*_max_ of 3.6 GHz were reported, and simple circuit demos, such as the megahertz MoS_2_ frequency mixer and common-source amplifier, were also demonstrated [26]. As has been done with exfoliated MoS_2_, optimized gate configuration was applied to CVD MoS_2_ transistors to achieve higher cut-off frequencies [27]. With optimized embedded gate structure, CVD monolayer MoS_2_ transistors with improved extrinsic *f*_T_ of 3.3 GHz and *f*_max_ of 9.8 GHz were fabricated. Although the RF performance of CVD MoS_2_ transistors was advanced with optimized device configuration, neither *f*_T_ nor *f*_max_ can reach the level of exfoliated MoS_2_ transistors, which severely limits their high-frequency applications. Bilayer MoS_2_ usually has higher carrier mobility and higher density of states, which in turn will result in superior performance over electronic devices based on monolayers [28,29,30]. In 2018, CVD bilayer MoS_2_ RF transistors with extrinsic *f*_T_ of 7.2 GHz and *f*_max_ of 23 GHz were demonstrated. For flexible high frequency transistors, extrinsic *f*_T_ and *f*_max_ of 2.7 and 2.1 GHz were obtained based on CVD monolayer MoS_2_ [11], and the extrinsic *f*_T_ and *f*_max_ of 4 and 9 GHz were obtained based on CVD bilayer MoS_2_ [31]. Although flexible MoS_2_ RF transistors based on CVD bilayer MoS_2_ have been demonstrated, there still many problems that need to be investigated. The reported flexible CVD bilayer MoS_2_ RF transistors only have one gate length. Its scaling behavior is still unexplored. Additionally, statistical analysis of MoS_2_ flexible RF transistors with different gate lengths needs more research, which is important to understand the uniformity of MoS_2_ RF transistors and helpful to acquire deep insight into the limited factor of large-scale high performance MoS_2_ RF transistors [32].

In this work, a systematic study of CVD bilayer MoS_2_ RF transistors on flexible substrates is presented. First, high-quality chemical vapor deposited bilayer MoS_2_ were grown on molten glass and transferred onto a Si_3_N_4_/PI substrate. The fabrication process of flexible high-frequency MoS_2_ transistors was presented. An extrinsic field-effect mobility of 5 cm^2^/Vs and a high *I*_on_/*I*_off_ ratio of 10^8^ were demonstrated with flexible bilayer MoS_2_ transistors. High-frequency measurements up to 10 GHz and statistical analysis were carried out for 14 MoS_2_ RF transistors with gate lengths of 0.3 μm, 0.6 μm, and 1 μm. Cut-off frequency and maximum oscillation frequency increased as the gate length was scaled down. Extrinsic *f*_T_ of 4 GHz and *f*_max_ of 10 GHz were achieved for the 0.3 μm transistors. Additionally, the transistors with larger gate length had better high-frequency performance uniformity. Finally, conversion gain of gigahertz MoS_2_ frequency mixer versus gate voltage, frequency and input power were systematically investigated.

## 2. Materials and Methods

### 2.1. Material Growth and Characterization

**Figure 1:**
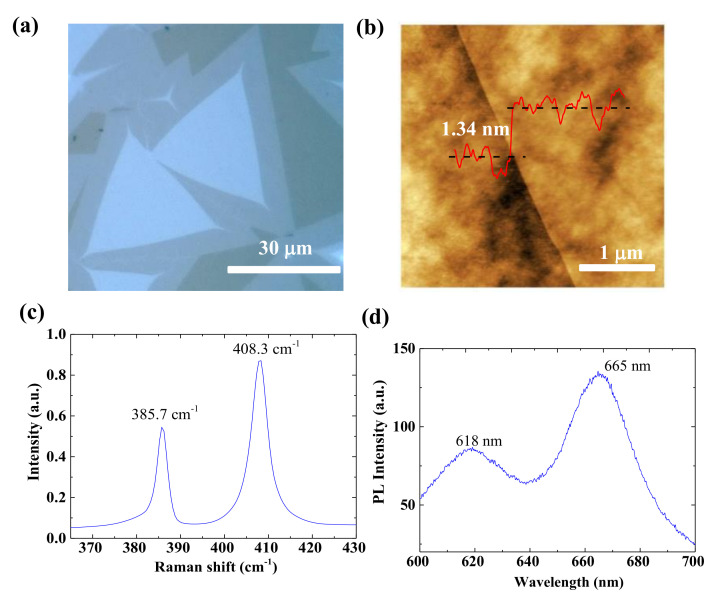
(**a**) Optical micrograph of chemical vapor deposition (CVD) bilayer MoS_2_ on molten glass. (**b**) Atomic force microscopy image of bilayer MoS_2_ on SiO_2_/Si substrates after transfer. Raman (**c**) and photoluminescence (PL) spectra (**d**) of high-quality CVD bilayer MoS_2_.

The bilayer MoS_2_ films were grown on molten glass substrates using sulfur powders and MoO_3_ powders as reaction precursors in a two-zone furnace. Then, an alumina crucible with 1.4 g sulfur powder and a quartz crucible with 2 mg MoO_3_ precursor and glass substrates were placed at the center of first and second heating zones, respectively. The distance between the MoO_3_ precursor and glass substrates was about 2 mm. The temperatures of the first and second zones were ramped to 230 °C and 830 °C, respectively. During the ramping and growth process, a 40 sccm Ar flow was introduced as the carrier gas, and the pressure within the quartz tube was controlled to 1 atm pressure. After a growth duration of 10 min, the furnace was turned off, and the system began to cool down. Figure 1a shows a typical optical microscopy of the bilayer; MoS_2_ with a triangular shape shows a highly uniform color contrast in the bilayer region, indicating homogenous thickness. The thickness and quality of the CVD bilayer MoS_2_ were then characterized by an atomic force microscope (AFM), Raman spectroscopy and photoluminescence analyses. Figure 1b shows the AFM image of the edge of the bilayer MoS_2_, and the corresponding height profile presents a thickness of 1.34 nm [33]. As shown in Figure 1c, after being transferred onto SiO_2_/Si substrates, the bilayer MoS_2_ shows typical Raman spectra with an *E*^1^_2g_ peak of 385.7 cm^−1^ and *A*_1g_ peak of 408.3 cm^−1^. The delta value between the *E*^1^_2g_ and *A*_1g_ peaks is 22.6 cm^−1^, which is consistent with previous reports of bilayer MoS_2_ [34,35,36]. The typical photoluminescence (PL) spectrum of the bilayer MoS_2_ is shown in Figure 1d, where the peaks at around 665 and 618 nm correspond to the A1 and B1 direct excitonic transitions at 1.86 and 2.01 eV, respectively [31,37]. What’s more, as shown in Figure S1, further elemental analysis of the CVD-grown MoS_2_ was studied by X-ray photoelectron spectroscopy (XPS). More details about material characterizations of CVD-grown MoS_2_ can be found in our previous works [31].

### 2.2. Device Fabrication

**Figure 2:**
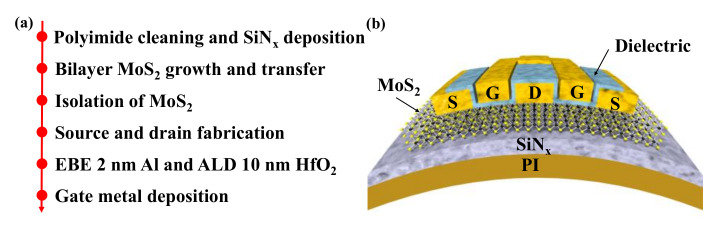
(**a**) Process flows and (**b**) schematic cross-section of the fabricated flexible bilayer MoS_2_ RF transistors.

**Figure 3:**
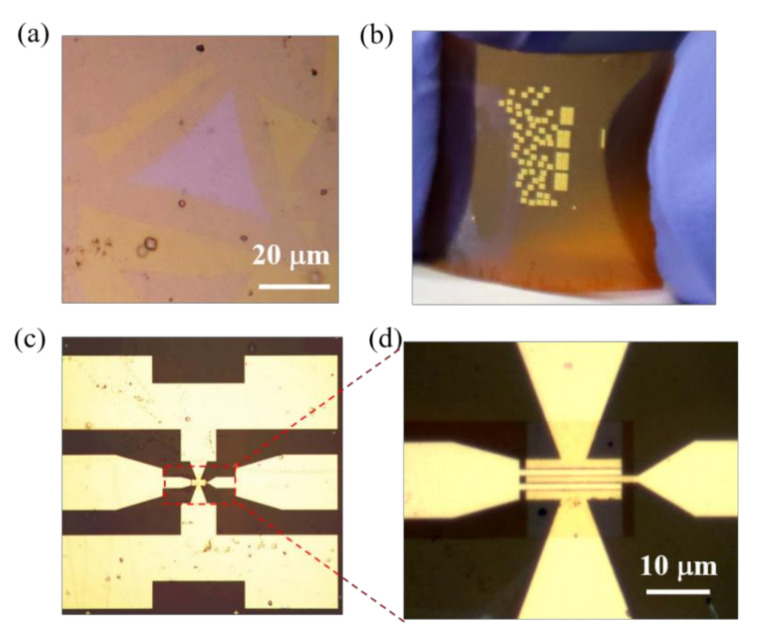
(**a**) The transferred bilayer MoS_2_ on polyimide substrates. (**b**) Optical images of the fabricated flexible MoS_2_ RF transistors. (**c**,**d**) Optical images of the flexible MoS_2_ RF transistor with ground-signal-ground (GSG) structure showing excellent alignment.

Figure 2 shows the fabrication process flows and device schematic of the bilayer MoS_2_ RF devices on flexible polyimide substrates. Prior to fabrication, the commercially available PI substrates (Dupont Kapton 500HN) were cleaned in acetone, isopropyl alcohol (IPA) and deionized water. To reduce the surface roughness, 100 nm of Si_3_N_4_ were deposited by plasma-enhanced CVD (PECVD). Then, bilayer MoS_2_ films were transferred onto polyimide substrates via the polymethyl methacrylate (PMMA)-assisted transfer method [23,25]. Different from conventional acidic or alkaline solutions as etchant, deionized water was utilized here for the hydrophobicity/hydrophilicity property of the as-grown MoS_2_/glass stack. As shown in Figure 3a, the high-quality triangle shape of bilayer MoS_2_ was well preserved, which is important for the fabrication of high-performance MoS_2_ transistors. After the transfer process, bilayer MoS_2_ films were patterned with an electron beam lithography (EBL) step and etched using O_2_/Ar plasma. Source and drain contact electrodes were formed by electron beam evaporation (EBE) with 20/60-nm Ni/Au metal stacks. To form a uniform top-gate dielectric, 2-nm Al was grown by EBE as a seed layer before the atomic layer deposition of HfO_2_. Finally, the two-fingers top-gate electrode of 20/60-nm Ni/Au was defined by EBL and deposited by EBE. The high-frequency bilayer MoS_2_ transistors on flexible PI substrates after fabrication are shown in Figure 3b,c. In this work, the MoS_2_ RF transistors are designed with different gate lengths of 0.3 μm, 0.6 μm, and 1 μm and the same gate width of 2 × 15 μm. Figure 3d shows an optical microscope of a device with a gate length of 1 μm, exhibiting the precise alignment of the gate structure to the source/drain area. From the theory of high-frequency electronics, both gate to drain/source capacitance *C*_gd_/*C*_gs_ and series resistance are critical factors in high-performance RF transistors. In this device design, there is no overlap between gate and source/drain electrodes to avoid excess *C*_gd_ and *C*_gs_. Additionally, the gate to source/drain access lengths *L*_gs_ and *L*_gd_ are minimized to decrease the series resistance.

## 3. Results and Discussion

### 3.1. DC Characterization

**Figure 4:**
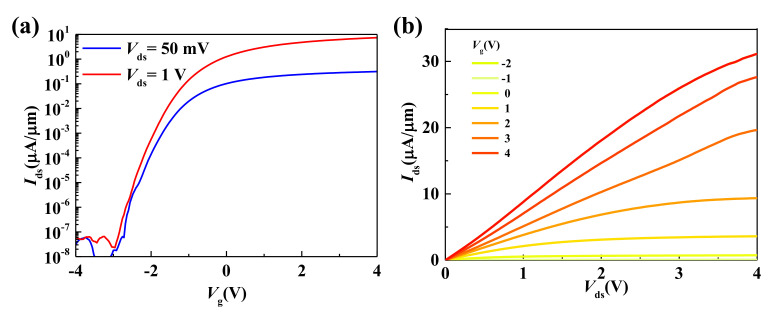
(**a**) Transfer characteristics at *V*_ds_ = 50 mV and 1 V. *I*_on_/*I*_off_ ratio are about 10^8^, making these devices ideal for ultra-low power applications. (**b**) Output characteristics of flexible CVD bilayer MoS_2_ transistors at various *V*_g_.

Figure 4a,b shows the measured transfer and output characteristics of CVD bilayer MoS_2_ transistors on polyimide substrates. A high *I*_on_/*I*_off_ ratio of 10^8^ is achieved, making these devices ideal for ultra-low power applications. The extrinsic low-field effect mobility of 5 cm^2^/Vs is extracted. Here, we note that the extracted mobility value is underestimated as there is a non-negligible contact resistance contribution to the total device resistance. The intrinsic mobility of our bilayer MoS_2_ on rigid substrates is calculated to be 36 cm^2^/Vs [31]. The degradation of mobility can be attributed to the increased surface roughness and poor thermal conductivity of organic flexible substrates. The output characteristics of bilayer MoS_2_ transistors on polyimide shows applicable current saturation. An on-current density of 30 μA/μm was demonstrated with the *L*_g_ = 1 μm device. For comparison, we also fabricated flexible monolayer MoS_2_ transistors on polyimide substrates. An extrinsic carrier mobility of 0.2 cm^2^/Vs and an on-current density of 1.2 μA/μm were obtained with the same gate length, showing the superiority of CVD bilayer MoS_2_ for flexible electronic applications.

### 3.2. RF Characterization

**Figure 5:**
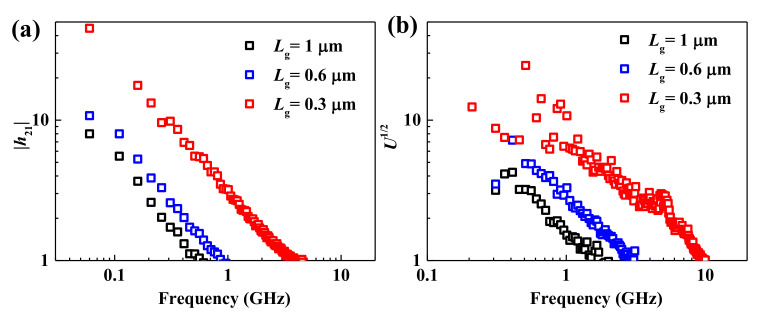
(**a**) Small-signal current gain |*h*_21_| versus frequency of flexible MoS_2_ transistors with gate lengths of 0.3 μm, 0.6 μm, and 1 μm. (**b**) The corresponding unilateral power gain versus frequency.

Cutoff frequency *f*_T_ and maximum oscillation frequency *f*_max_ are commonly used to characterize the high-frequency performance of RF transistors. *f*_T_ corresponds to the frequency where the short-circuit current gain becomes unity. From the small signal equivalent circuit of the transistor, *f*_T_ can be described using

(1) $$f_{T}=\frac{g_{m}}{2\pi }*\frac{1}{\left(C_{\mathrm{gs}}+C_{\mathrm{gd}})[1+g_{\mathrm{ds}}(R_{s}+R_{d})]+C_{\mathrm{gd}}g_{m}(R_{s}+R_{d}\right)}$$

where *C*_gs_ is the gate to source capacitance, *C*_gd_ is the gate to drain capacitance, *g*_m_ is the transconductance, *g*_ds_ is the output conductance, and *R*_s_ and *R*_d_ are the source and drain series resistances, respectively. *f*_max_ correspond to the frequency where the unilateral power gain becomes unity. *f*_max_ can be described using

(2) $$f_{\max}=\frac{f_{T}}{2\sqrt{g_{\mathrm{ds}}(R_{s}+R_{d})+2\pi f_{T}C_{g}R_{g}}}$$

where *R*_g_ is the gate resistance, which can be reduced through the increase of gate metal area and thickness. To evaluate the high-frequency performance of the CVD bilayer MoS_2_ transistors, standard on-chip S-parameter measurements up to 10 GHz were performed. Figure 5a,b show the as-measured extrinsic small-signal current gain (|*h*_21_|) and Mason’s unilateral power gain (*U*) as a function of frequency for the CVD bilayer MoS_2_ transistors. An extrinsic *f*_T_ of 4 GHz and *f*_max_ of 10 GHz were achieved where, as shown in Table 1, the *f*_max_ is the highest extrinsic maximum oscillation frequency among flexible MoS_2_ RF transistors [9,11], demonstrating the potential of bilayer MoS_2_ for large-scale, high-performance RF applications. What’s more, *f*_max_ is also comparable to MoS_2_ transistors on rigid substrates with the same gate length [31]. This can be attributed to the decreased high-frequency parasitic effect in insulating polyimide substrates, although the roughness and poor thermal conductivity of polyimide substrates degrade the DC transport performance of the MoS_2_ transistors [12].

**Figure 6:**
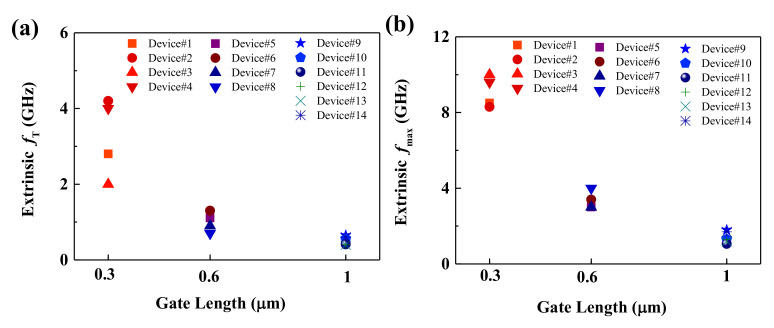
Extrinsic *f*_T_ and *f*_max_ of 14 flexible MoS_2_ RF transistors. (**a**) Extrinsic *f*_T_ as a function of gate length. (**b**) Extrinsic *f*_max_ as a function of gate length.

Figure 6, displaying the devices’ data, gives some insight into the statistical analysis and scalability of CVD bilayer MoS_2_ RF transistors on flexible PI substrates. The extrinsic *f*_T_ and *f*_max_ of 14 MoS_2_ devices with different gate lengths are plotted in Figure 6a,b, respectively. Both *f*_T_ and *f*_max_ increase as the gate length decreases. Additionally, through gate length scaling down, it is possible to further improve the *f*_T_ and *f*_max_. Variations of *f*_T_ and *f*_max_ within devices of the same gate length can be observed, especially for transistors with a gate length of 0.3 μm. Here, we attribute these variations to the varied alignment of gate and source/drain electrodes in the EBL process of short gate length transistors. Since flexible organic substrates are not conductive and easily result in deformation, not all devices in this work could realize the perfect alignment of gate and source/drain electrodes, as shown in Figure 3d. Short gate length RF transistors are more prone to uniformity problems. Therefore, the contact resistance, substrate roughness and fabrication process are still critical limitations of flexible bilayer MoS_2_ RF transistors. It should be pointed out that MoS_2_ transistors with 1T phase electrodes exhibiting contact resistance of 200–300 Ω∙μm have been demonstrated [38]. MoS_2_ polymorphs with diverse electrical properties and their applications in high-frequency nanoelectronics are fascinating [18,38,39,40,41,42,43] and require further investigation.

**Figure 7:**
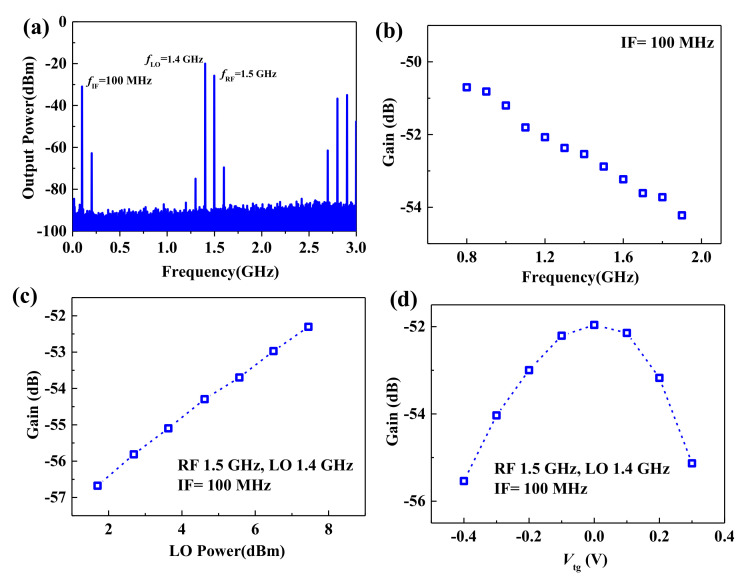
Gigahertz flexible MoS_2_ mixer. (**a**) Output frequency spectrum of the flexible MoS_2_ mixer. (**b**) Conversion gain of MoS_2_ mixer versus input frequency. (**c**) Conversion gain of MoS_2_ mixer versus local oscillation (LO) power. (**d**) Conversion gain of MoS_2_ mixer versus gate voltage.

Although flexible MoS_2_ mixers have been demonstrated in previous work [11,31], the frequency response and gate bias voltage dependence of flexible MoS_2_ mixers have not been reported. In this work, active mixers based on flexible bilayer MoS_2_ transistors were demonstrated and systematically researched. Mixer measurements were carried out at room temperature with an RF input frequency of 1.5 GHz and local oscillation (LO) frequency of 1.4 GHz. Figure 7a shows the measured IF output signal (0.1 MHz) using a signal analyzer. The conversion gain versus the input frequency is plotted in Figure 7b. When the input signal powers and *f*_IF_ of 100 MHz are the same, conversion gain decreases as frequency increase from 0.8 to 1.9 GHz. Figure 7c shows the conversion gain versus LO power. A conversion gain of −52.3 dB could be achieved with *f*_RF_ of 1.5 GHz. For the flexible active MoS_2_ mixer, as shown in Figure 7d, gate bias voltage is important to achieve the maximum conversion gain. This is because DC transconductance, the same as cutoff frequency and maximum oscillation frequency, has a strong dependence on gate bias voltage.

## 4. Conclusions

In this work, we constructed high-frequency transistors and gigahertz mixers on flexible polyimide substrates based on CVD bilayer MoS_2_, and their high-frequency performance was systematically assessed. Record extrinsic *f*_max_ as high as 10 GHz have been demonstrated with 0.3 μm devices. The scaling behavior of MoS_2_ RF transistors on flexible polyimide substrates was studied, and the extrinsic *f*_T_ and *f*_max_ were increased as gate length decreased, showing the potential for further improvement through decreasing the gate lengths. Statistical analysis of 14 flexible MoS_2_ RF transistors with different gate length showed RF performance variation in short gate length MoS_2_ transistors. We systematically studied the dependence of flexible MoS_2_ mixer conversion gain on gate bias voltage, *f*_RF_ and LO power, addressing the importance of *V*_tg_, LO power and *f*_RF_ on high-performance flexible MoS_2_ mixers. Our results advance the achieved maximum oscillation frequency of flexible CVD MoS_2_ transistors and represent a step towards high-performance flexible MoS_2_ wireless communications systems.

## Figures and Tables

**Table 1:** Comparison of flexible high-frequency transistors based on 2D MoS_2._

MoS_2_	Substrate	*L*_g_ (nm)	*f*_T,extrinsic_ (GHz)	*f*_max,extrinsic_ (GHz)	References
Exfoliated	PI	68	4.7	5.4	[9]
CVD	PI	500	2.7	2.1	[11]
CVD	PI	300	4	9	[31]
CVD	PI	300	4	10	This Work

## Data Availability

The data that support the findings of this study are available from the corresponding author upon reasonable request.

## Supplementary Materials

The following are available online at https://www.mdpi.com/article/10.3390/mi12040451/s1, Figure S1: (a) The XPS spectra of the Mo 3d state, (b) S 2s state.
