# Supplementary material for: High-Performance CVD Bilayer MoS2 Radio Frequency Transistors and Gigahertz Mixers for Flexible Nanoelectronics
Source: Micromachines (Basel). 2021 Apr 16;12(4):451. doi: 10.3390/mi12040451 (PMC8072592; doi:10.3390/mi12040451)
Supplement: Supplementary file 1 [file micromachines-12-00451-s001.zip › micromachines-1180361-supplementary.pdf]

micromachines

MDPI

Article

High-Performance CVD Bilayer MoS_2 Radio Frequency Transistors and Gigahertz Mixers for Flexible Nanoelectronics

Qingguo Gao [1,2], Chongfu Zhang [1,2,*], Kaiqiang Yang [1], Xinjian Pan [1], Zhi Zhang [1], Jianjun Yang [1], Zichuan Yi [1], Feng Chi [1] and Liming Liu [1]

[1] School of Electronic Information, University of Electronic Science and Technology of China, Zhongshan Institute, Zhongshan 528402, China; gqgemw@163.com(Q.G.); 201811022515@std.uestc.edu.cn (K.Y.); xinjpan@163.com (X.P.); zz001@zsc.edu.cn (Z.Z.); sdyman@uestc.edu.cn (J.Y.); yizichuan@zsc.edu.cn (Z.Y.); chifeng@semi.ac.cn (F.C.); limingliu@uestc.edu.cn (L.L.)

[2] School of Information and Communication Engineering, University of Electronic Science and Technology of China, Chengdu 611731, China

* Correspondence: cfzhang@uestc.edu.cn

S1 XPS analysis

Figure S1 shows the X-ray photoelectron spectroscopy (XPS) of the CVD grown MoS_2. In Fig. S1(a), the XPS spectra shows two peaks at 230.3 and 233.4 eV, corresponding to the Mo $3d_{5/2}$ and Mo $3d_{3/2}$ state, respectively. As shown in Figure S1(b), the binding energy for S $2p_{3/2}$ and S $2p_{1/2}$ are 163.2 and 164.4 eV. The data of Mo 3d, and S 2p states are in agreement with the reported data of MoS_2 samples with 2H crystalline structure[1,2]. The atomic concentration of Mo (3d) to S (2p) in MoS_2 was estimated to be 34.36 to 65.64, respectively.

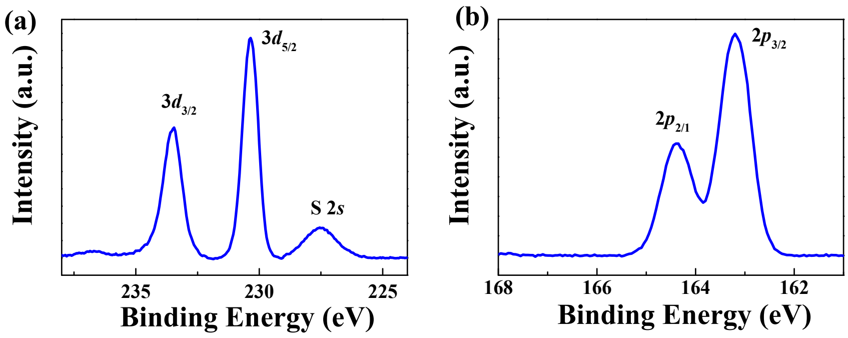

Figure S1. (**a**) The XPS spectra of the Mo 3d state, (**b**) S 2s state.

References

1. Lee, Y., Lee, J., Bark, H., Oh, I.-K., Ryu, G.H., Lee, Z., Kim, H., Cho, J.H., Ahn, J.-H., Lee, C., Synthesis of wafer-scale uniform molybdenum disulfide films with control over the layer number using a gas phase sulfur precursor, *Nanoscale* **2014**, *6*, 2821–2826.
2. Park, S.J., Chu, D., Kim, E.K., Controllable Growth of Single Layer MoS_2 and Resistance Switching Effect in Polymer/MoS_2 Structure, *Appl. Sci. Converg. Technol.* **2017**, *26*, 129–132.
